# Research Progress on the Preparation and Application of Decellularized Tendons

**DOI:** 10.3390/cimb47040251

**Published:** 2025-04-06

**Authors:** Jing Li, Mingxing Wen, Sujuan Zhang, Lingfei Du, Xin Fan, Hao Liang, Hong Wang, Jing Sun, Yuchun Ding, Liangpeng Ge, Jideng Ma, Jinwei Zhang

**Affiliations:** 1 Chongqing Academy of Animal Sciences, Chongqing 402460, China; lijing09002022@163.com (J.L.); fanxin.5@163.com (X.F.); lianghaowd@163.com (H.L.); 18811017313@163.com (H.W.); sunjing85026@163.com (J.S.); dingyuchun@yeah.net (Y.D.); geliangpeng1982@163.com (L.G.); 2 Sichuan Provincial Key Laboratory of Exploration and Innovative Utilization of Livestock and Poultry Genetic Resources, Sichuan Agricultural University, Chengdu 611130, China; wenmx0126@163.com; 3 National Center of Technology Innovation for Pigs, Chongqing 402460, China; 15236606571@163.com (S.Z.); lingfeidu0329@163.com (L.D.); 4 Ministry of Agriculture Key Laboratory of Pig Sciences, Chongqing Key Laboratory of Pig Sciences, Chongqing 402460, China

**Keywords:** tendons, decellularization, structural components, applications

## Abstract

Tendons connect animal skeletons to skeletal muscles, playing a crucial role in weight-bearing and maintaining motor functions. After decellularization, tendon extracellular matrix (tECM) retains the physicochemical characteristics similar to those of native tendons. This has made tECM a promising biomaterial in the fields of tissue engineering and regenerative medicine in recent years. This paper summarizes the origin, structure, and ECM components of animal tendons, reviews decellularization methods, and discusses recent advancements in the research and applications of decellularized tendons. Furthermore, it explores future development trends of xenogeneic decellularized tendon materials, aiming to provide a reference for fundamental research and the development of biomaterials related to decellularized tendons.

## 1. Introduction

Tendons are dense connective tissues that connect bones and skeletal muscles, playing a critical role in maintaining body posture as well as the integrity and function of the musculoskeletal system by buffering and transmitting forces. Tendon injuries are among the most common conditions affecting the musculoskeletal system, significantly impairing patients’ mobility [1,2]. Due to the intrinsic hypovascularization of tendons, the process of regeneration following injury is characterized by its slowness and frequent association with fibrous scar formation. Moreover, it is challenging to restore the tendon’s original mechanical properties, even following surgical intervention. Post-surgically, the modulus of elasticity of the tendon is typically reduced to 60–70% of its pre-injury value, and there is a 20–40% risk of rerupture [3]. Current tendon repair techniques have evolved from a focus on purely structural restoration to more comprehensive approaches that consider biomechanical strength, minimally invasive methods, and functional rehabilitation. The employment of multi-strand sutures, the utilization of minimally invasive access methods, and the implementation of early activity protocols have become the prevailing standard of practice. The integration of novel materials and biologics represents a significant potential for further advancements in the domain of tendon repair [4,5,6].

In the domain of clinical repair, decellularized tendon materials are garnering increasing appreciation for their distinctive biological advantages. Through the optimization of a combination of chemical treatments, physical methods and enzyme digestion techniques, a combination of protocols can effectively remove cellular components, while retaining intact the natural orientation structure of the collagen fiber bundles, While preserving collagen structure, key ECM components and growth factors [7,8,9]. This ECM microenvironment, which is highly biomimetic, has been shown to promote directional migration of tendon stem cells and tendonogenic differentiation. In addition, it has been demonstrated to modulate cell-mechanical signal transduction through retained integrin binding sites. This property is significantly superior to the biological inertness of synthetic materials [10,11].

Hydrogels are materials with high water content, elasticity, and bioactivity, which not only facilitate the diffusion of nutrients and bioactive factors but also serve as carriers for cells and drugs, minimizing immune reactions and providing a novel approach for tendon therapy [12,13,14,15]. In comparison with conventional hydrogel systems, decellularized tendon materials exhibit distinctive structural-functional advantages. The natural fiber bundle structure of decellularized tendons guides the directional alignment of cells, a property often lacking in hydrogels due to their homogeneous structure. Furthermore, decellularized tendons possess a modulus of elasticity that closely resembles that of natural tendons. The modulus of elasticity of a decellularized tendon is more closely aligned with that of a natural tendon than with that of conventional hydrogel, which is 2–4 orders of magnitude higher. This suggests that the former maintains the mechanical stability of the repair process more effectively [7,16,17]. In the most recent study, the composite of decellularized tendon powder and temperature-sensitive hydrogel not only maintains the bioactivity of the ECM but also improves the injectability of the material, thereby increasing the mechanical recovery rate in a rabbit Achilles tendon defect model [16,17].

In recent years, 3D printing technology has been applied to tendon tissue engineering, enabling precise control over composition, spatial distribution, and structure to fabricate biocompatible, absorbable, and biodegradable biomaterials [18,19,20,21,22]. The integration of 3D printing technology with decellularized tendons represents a significant advancement, circumventing the constraints imposed by conventional scaffolds. The synergistic printing of decellularized ECM bioink with fused PCL has enabled the fabrication of multilayered scaffolds with a hierarchical pore structure. The yield strength of these scaffolds approximates that of natural tendons while retaining the biological signals that promote tendon differentiation [8,23]. This hybrid printing strategy yielded a substantial enhancement in collagen fiber orientation when compared to single material systems [23].

This paper summarizes the structure, and ECM components of animal tendons, as well as various decellularization methods. It also reviews recent research progress on the applications of decellularized tendons, aiming to provide a reference for the preparation and application of xenogeneic decellularized tendon biomaterials.

## 2. Extracellular Matrix Components and Structure of Tendons

### 2.1. Extracellular Matrix Components of Tendons

The tendon ECM mainly consists of collagen, proteoglycans, glycoproteins, and elastin. The ECM not only provides a microenvironment for cell survival but also maintains tissue morphogenesis, differentiation, and homeostasis [24].

#### 2.1.1. Collagen

Collagen accounts for approximately 70% of the dry weight of tendons, with type I collagen being the predominant type. Type I collagen fibers provide tendons with tensile strength and the ability to undergo mechanical deformation [25]. In addition to type I collagen, tendons also contain type III, type V, and type XI collagen. Type III collagen is highly expressed during embryonic development and plays a role in regulating the diameter of collagen fibrils [26]. Type V collagen is located at the core of collagen fibers and is involved in the tendon development process. Type XI collagen is widely expressed during tendon development and plays a role in regulating collagen fiber assembly [27]. Additionally, tendons contain small amounts of non-fibrillar collagens. Type VI collagen is typically enriched around cells, and its absence can lead to abnormal fibril alignment and reduced mechanical performance. Type XII collagen has a stabilizing effect on collagen fibers. Type XIV collagen regulates fibril diameter and acts as a molecular bridge between collagen fibrils and other matrix molecules during tendon development [28,29] (Table 1).

#### 2.1.2. Proteoglycans

Proteoglycans (PGs) are distributed among tendon fibrils, collagen fibers, and fascicles. PGs are composed of a protein core covalently linked to one or more glycosaminoglycan (GAG) side chains. GAGs are mucopolysaccharides with a negative charge, capable of attracting water molecules and existing in a gel-like state [30,31]. Tendon PGs mainly include small leucine-rich proteoglycans (SLRPs) and large aggregating proteoglycans (LAPs). SLRPs can be divided into two classes: Class I, which includes decorin and biglycan (BGN), and Class II, which includes fibromodulin (FMOD) and lumican (LUM) [32]. Decorin is the most abundant SLRP in tendons. It binds to specific regions of collagen fibers via non-covalent interactions, restricting their lateral growth [33]. BGN competes with decorin for collagen fibril binding sites, maintaining the structure of collagen fibrils while regulating collagen fiber formation and ECM assembly. FMOD is involved in tendon collagen synthesis, cell proliferation, and matrix remodeling [34]. LUM can induce an increase in interfibrillar spacing and a reduction in fibril diameter [35]. LAPs include aggrecan, lubricin, and versican. Aggrecan increases the water content of tendons, provides compressive resistance, and slows the formation of collagen fibers. Lubricin is primarily located on the tendon surface, where it lubricates the tendon and prevents cell adhesion. Versican is mainly distributed in the interfascicular matrix (IFM) and supports the cell shape changes required for cell proliferation and migration [30,36] (Table 1).

#### 2.1.3. Glycoproteins

Cartilage oligomeric matrix protein (COMP) is the most abundant glycoprotein in tendons. The five subunits of COMP bind to five collagen molecules, facilitating interactions between COMP and other matrix proteins to form a network structure that provides mechanical support to tendons [37]. Tenascin-C (TNC) is a hexameric protein that regulates the adhesion of tendon cells to the ECM, promotes fibroblast proliferation and migration, facilitates tendon repair after injury, and plays a role in the differentiation of fibroblasts into tendon cells. TNC expression is elevated during wound healing, pathological conditions, or under high mechanical stress [38]. Fibronectin (FN) is a dimer composed of subunits linked by disulfide bonds. It binds to collagen, heparan sulfate, and integrin receptors on the surface of tendon cells, playing a crucial role in supporting and regulating the ECM [39]. Elastin is a highly elastic cross-linked structural protein that provides viscoelasticity to tendons and facilitates collagen fiber sliding. Elastic fibers, composed of elastin, help maintain tendon shape and elasticity, with the ability to withstand tensile and compressive forces without easily breaking [40,41].

The spatial distribution and cross-linking status of tendon ECM components are pivotal in determining their biomechanical properties. Moreover, these factors directly impact the recellularization efficiency and mechanical property retention of decellularized scaffolds. The maintenance of the natural ratio and three-dimensional structure of these components during the subsequent decellularization process is pivotal to the technology [42,43] (Table 1).

**Table 1:** The main components of the tendon extracellular matrix.

Variety	Type	Function	References
Collagen	Type I Collagen	Provides tensile strength and mechanical deformation ability to tendons	[25]
	Type III Collagen	Regulates collagen fibril diameter	[26]
	Type V Collagen	Involved in tendon development	[27]
	Type XI Collagen	Regulates collagen fiber assembly	[28,29]
	Type VI Collagen	Absence leads to abnormal fibril alignment and reduced mechanical performance	
	Type XII Collagen	Stabilizes collagen fibers	
	Type XIV Collagen	Regulates collagen fibril diameter	
Proteoglycan	Glycosaminoglycans	Negatively charged, attracts water molecules	[31]
	Decorin	Restricts lateral growth of collagen fibers	[33]
	Biglycan	Maintains collagen fibril structure and regulates collagen fiber formation and ECM assembly	[34]
	Fibromodulin	Involved in collagen synthesis, cell proliferation, and matrix remodeling	
	Lumican	Induces increased interfibrillar spacing and reduced fibril diameter	[35]
	Aggrecan	Provides compressive resistance to tendons and slows collagen fiber formation	[30,36]
	Lubricin	Induces increased interfibrillar spacing and reduced fibril diameter	
	Versican	Promotes collagen fiber formation, regulates collagen compaction and reorganization	
Glycoprotein	COMP	Provides mechanical support and stability to tendons	[37]
	Tenascin-C	Regulates tendon cell adhesion to ECM, fibroblast proliferation, migration, differentiation, and repair	[38]
	Fibronectin	Supports and regulates ECM	[39]
Elastin		Provides viscoelasticity to tendons and facilitates collagen fiber sliding	[40]

### 2.2. Hierarchical Structure of Tendons

**Figure 1:**
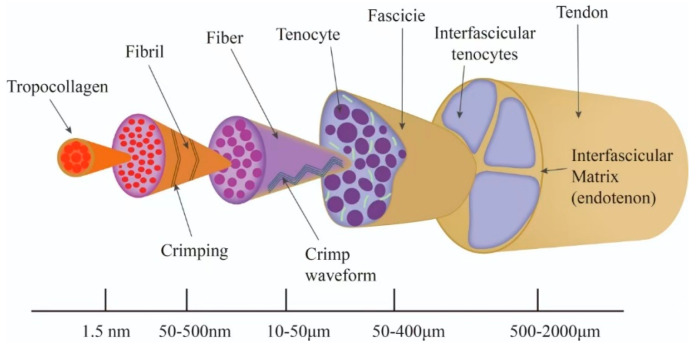
Schematic diagram of tendon structural hierarchy. Collagen molecules initially aggregate to form collagen protofibrils. These collagen protofibrils then aggregate to form collagen fibrils. Subsequently, collagen fibrils combine to form fascicles that increase in diameter.

The hierarchical structure of the tendon is characterized by a high degree of order and multiscale organization, with its structure exhibiting a step-by-step assembly process. The fundamental structural element of tendon is the type I collagen molecule (three-stranded helical structure), which establishes a robust network through lysyl oxidase-mediated intermolecular crosslinks, thereby determining the tensile strength of tendon [44]. Collagen molecules form protofibrils through a process of self-assembly, and their surface is covered with proteoglycans (e.g., core proteoglycan decorin) and glycosaminoglycans (GAGs) [45,46]. These components regulate the diameter and spacing of protofibrils. Glycoproteins, such as fibronectin, have been observed to promote the longitudinal alignment of protofibrils through adhesion [47]. Protofibrils are further cross-linked by fibronectin to form collagen fibers. Energy-storing tendons (e.g., the Achilles tendon) have significantly higher levels of GAGs on the fiber surface than positional tendons, and this difference adapts to the different mechanical demands by modulating the transfer of shear stresses between the fibers [48]. Collagen fibers form tendon bundles in a highly ordered parallel arrangement. Intra-bundle fibers are laterally cross-linked by glycoproteins, including fibronectin and laminin, while the inter-bundle matrix is rich in glycosaminoglycans, such aschondroitin sulfate, which modulate the resistance to inter-fiber sliding by hydration [49]. Tendon bundles form a multilevel helical structure through proteoglycan/glycosaminoglycan complexes, and this hierarchical anisotropy gives tendons both high tensile strength and toughness [50,51] (Figure 1).

## 3. Tendon Decellularization Methods

Decellularization is defined as a bioprocess that removes cellular components from a tissue or organ by means of chemical, physical, or enzymatic treatments, while ensuring the retention of the maximum amount of ECM structure, biologically active components and mechanical properties [52]. The primary objective is to eradicate immunogenic cellular remnants (e.g., DNA) while preserving the three-dimensional structural and functional characteristics of the ECM. This approach ensures the provision of an optimal biological scaffold for tissue engineering applications [53].

### 3.1. Physical Methods

Physical decellularization methods involve the use of ultrasound, repeated freeze–thaw cycles, and mechanical forces to physically disrupt cell membranes, leading to cell fragmentation and lysis. The primary benefit of this approach is the elimination of chemical residues. However, there is a potential risk of selective loss of ECM components. The cavitation effect refers to the localized high temperature, high pressure, and shock waves generated by strong ultrasound, which can destroy surrounding cells. Ultrasound at specific frequencies can loosen tendon structures, facilitating the penetration of decellularization agents [8]. In dense tissues (e.g., tendons), a combination of frequency modulation is required to balance cell clearance with collagen fiber damage [54]. Repeated freeze–thaw cycles utilize the formation of ice crystals during multiple freezing and thawing processes to disrupt cell membrane structures, thereby achieving decellularization [55]. The freeze–thaw process can have varying degrees of impact on the ECM structure, so it is crucial to strictly control the rate of temperature change during the procedure to prevent severe damage to the ECM caused by ice crystals [56]. Mechanical forces involve the use of external forces to disrupt cell membranes [57]. The material is especially well-suited to shallow or monolayer cellular structures. However, it is important to note that mechanical forces may disrupt the microstructure of the ECM (e.g., elastic fiber breakage), which can lead to a decrease in the modulus of elasticity or compressive strength of the scaffold after decellularization (Table 2).

The most conservative current physical approach is to combine automated decellularization equipment with programmed freeze–thaw cycles. This reduces manual errors and retains more ECM functional proteins [58].

**Table 2:** Summary of decellularized methods.

Decellularized Methods	Specific Method	Decellularization Principle	References
Physical methods	Ultrasound	Loosens tendon structure, facilitating reagent penetration	[8]
	Repeated freeze–thaw	Ice crystals disrupt cell membranes	[55]
	Mechanical stirring	Mechanical forces disrupt cell membranes	[57]
Chemical methods	Acid	Perforates tendon surfaces to facilitate reagent penetration; dissolves cell membranes	[59]
	Detergents (SDS, Triton X-100, TBP)	Dissolves cell membranes, disrupts DNA-protein interactions	[60]
	EDTA	Disrupts adhesion between ECM components	[61]
	Hypertonic/Hypotonic solutions	Uses osmotic pressure to disrupt cells	[62]
Biological methods	Trypsin	Digests proteins and peptides, disrupts extracellular matrix around collagen fibers	[63]
	DNase/RNase	Removes residual DNA and RNA left after cell lysis	[64]

### 3.2. Chemical Methods

Common chemical reagents include acids, detergents, hypertonic and hypotonic solutions, as well as the chelating agent ethylenediaminetetraacetic acid (EDTA). The utilization of chemical reagents can facilitate efficient decellularization; however, this process carries inherent risks, including the potential irreversible loss of ECM and the presence of chemical residues. Acids perforate the tendon surface to disrupt its dense structure and dissolve cell membranes, releasing intracellular contents [59]. Ionic detergent, sodium dodecyl sulfate (SDS), can dissolve cell and nuclear membranes, leading to cell lysis, but can also cause some degree of ECM damage. Non-ionic detergent, Triton X-100, can disrupt lipid–protein interactions without affecting protein–protein connections, but it partially removes GAGs. Zwitterionic detergent, tri-n-butyl phosphate (TBP), can disrupt protein–protein interactions with minimal impact on tendon mechanical properties but reduces collagen content [60]. Disruption of cell-ECM adhesion is achieved by EDTA through its capacity to chelate calcium (Ca^2+^) and magnesium (Mg^2+^) ions, and is commonly used in decellularization buffer solutions for tendons [61]. Hypertonic and hypotonic solutions disrupt cells by osmotic pressure. To achieve better penetration, hypertonic and hypotonic solutions are often alternated over multiple cycles, which helps to remove cellular residues [62]. To reduce the toxicity of chemical reagents, thorough washing is usually required to remove residual reagents from the tendon tissue (Table 2).

### 3.3. Biological Methods

Biological decellularization, a process that employs enzymes to digest cell nuclei and cell membranes, offers the advantage of molecular specificity. However, it is encumbered by issues pertaining to deep penetration and the retention of activity. Typically, it is employed in conjunction with chemical reagents or physical decellularization techniques. Commonly used enzymes include trypsin and nucleases (DNase and RNase). Trypsin, used at 37 °C, digests proteins and peptides, disrupting the extracellular matrix surrounding collagen fibers and creating microchannels to facilitate the penetration of decellularization agents into deeper regions of the tendon, separating cells from the ECM (Table 2).

Nucleases are less effective at removing DNA from large, densely structured tendons. To address this issue, tendon tissue can be sliced into thin sections prior to decellularization or treated with nucleases after decellularization [63,64]. 

### 3.4. Combined Methods

A single decellularization method is often insufficient for complete cell removal. The use of only chemical or physical decellularization methods may induce the release of intracellular components into the surrounding ECM, necessitating more effective washing steps to remove cellular debris [65]. Combining different decellularization methods with tendons from different species and anatomical locations can better achieve effective decellularization while preserving the tendon’s internal ultrastructure, various biochemical components, and favorable biomechanical properties (Table 3).

**Table 3:** Preparation of decellularized tendons in different species.

Species	Tendon Type	Decellularization Protocol	Result	References
Pig	Patellar tendon	Tris buffer (containing alanine and EDTA), SDS, PBS washing, nuclease solution, PBS washing, ultrasound at different intensities	Under ultrasound conditions (360 W, 1 s pulse, 1 min), the tendon displayed a porous structure with no significant effects on biochemical composition or biomechanics	[66]
Horse	Superficial digital flexor tendon	Automated freeze–thaw cycles (freezing machine) and manual freeze-thaw cycles (liquid nitrogen 2 min, 37 °C 10 min, 5 cycles), distilled water, Tris buffer (1% Triton X-100)	No significant difference between automated and manual freeze–thaw cycles; effective for large tendons	[56]
Rabbit	Flexor tendon and semitendinosus tendon	Six protocols tested: 1% Triton-X 100, 0.5% SDS, 1% TBP, 1% Triton-X 100 + 0.5% SDS, 1% TBP + 0.5% SDS, 1% TBP + 1% Triton-X 100, followed by distilled water, nuclease, and EDTA	Treatment with 1% TBP + 0.5% SDS achieved complete cell removal, with histology and biomechanics similar to native tendon tissue	[63]
Cattle	Achilles tendon	Tissue cut into 0.6 mm slices, freeze–thaw cycles (liquid nitrogen 1 min, 37 °C min, 5 cycles), PBS washing, nuclease solution, α-galactosidase, PBS washing	DNA and α-gal epitopes effectively removed, with good preservation of collagen fibers and chondroitin sulfate characteristics	[67]
Beagle	Achilles tendon	Tissue cut into 40 mm slices, PBS washing, repeated freeze–thaw (liquid nitrogen 2 min, 37 °C 10 min), PBS washing, nuclease solution, PBS washing	Repeated freeze–thaw combined with nuclease treatment for twelve hours achieved complete decellularization, with ultrastructure well-preserved	[68]
Goat	Flexor tendon	Tissue cut into 2 mm slices, hypotonic solution (4 freeze–thaw cycles), hypertonic solution, SDS, Triton-X 100, ultrapure water washing	DNA content reduced by over 95%. Post-decellularization, fiber morphology was intact, and collagen content showed no significant difference from native tendon tissue	[69]
Macaque	Achilles tendon	Tissue cut into 2 cm slices, repeated freeze–thaw, cryosectioned into 300 µm slices, nuclease treatment, PBS washing	H&E staining, DAPI staining, and DNA quantification confirmed the effectiveness of decellularization in macaque Achilles tendons	[16]

## 4. Cross-Species Characterization Differences in Tendon Properties

### 4.1. Species-Specific Differences in ECM Components

Tendons from different species exhibit significant differences in collagen subtype distribution (e.g., ratio of type I to type III collagen), proteoglycans (e.g., core proteoglycans, disaccharide chain proteoglycans), and glycosaminoglycans (GAGs) content [70]. For instance, the collagen cross-link density and elastic modulus of the bovine tendon are significantly higher than those of the rodent tendon, whereas the GAGs content of the porcine tendon is closer to that of the human tendon. This directly affects the bioactivity and immunocompatibility of the decellularized scaffolds [11,71]. Furthermore, the distribution pattern of fibronectin and laminin, which is specific to human tendons, may not be fully reproduced in rodent models [48,72]. For this reason, species with high sequence homology of ECM proteins (e.g., porcine) are preferred for xenotransplantation in order to preserve key signaling molecules [71,73].

### 4.2. The Dependence of Biomechanical Properties on Species

The stress relaxation, creep behavior and ultimate tensile strength (UTS) of tendons are species-specific. To illustrate this point, the UTS of the human Achilles tendon (~100 MPa) is significantly higher than that of the mouse (~30 MPa), while the viscoelasticity of the porcine tendon is closer to that of the human [74]. The selection of donor tissue must be tailored to the mechanical demands of the intended application. For instance, the bovine Achilles tendon is well-suited for weight-bearing site repair due to its high stiffness and fatigue resistance [71,74], while the rabbit flexor tendon, with its superior elasticity, is more appropriate for hand tendon repair. It is noteworthy that the hyperlipidemic pig model demonstrated that ECM lipid deposition reduces collagen fiber slippage, resulting in the attenuation of scaffold mechanical properties following decellularization. This suggests a limitation of animal models in the study of metabolic diseases [71,75].

### 4.3. Biocompatibility Influencing Factors

The microstructure (e.g., fiber bundle arrangement, proportion of interfibrillar matrix) of tendons varies considerably between species. For example, rodent tendons have less interfibrillar matrix, whereas tendons from large mammals (e.g., cows, pigs) more closely resemble the hierarchical structure of human tendons [70,76]. This difference affects the ability of the three-dimensional microenvironment of the scaffold to transmit mechanical signals to host cells after decellularization. Scaffolds that mimic the fiber arrangement of healthy tendons were shown to promote directional cell migration and ordered ECM deposition [70,77]. Meanwhile, differences in the immunogenicity of ECM proteins between species need to be specifically evaluated. Despite the fact that mammalian ECM proteins demonstrate more than 80% homology [71], species–specific antigens have the potential to induce post-transplant immune rejection [73]. For instance, the presence of chondroitin sulfate residues from bovine-derived decellularized scaffolds has been shown to activate the human macrophage TLR4 pathway, whereas type I collagen α2 chain variants from equine tendons have been observed to interfere with integrin signaling [48,73]. In contrast, human- or porcine-derived ECMs are likely to be safer due to their higher degree of homology [76]. Furthermore, cross-species decellularization has been shown to retain specific glycosylation-modified proteins (e.g., fibronectin), which require optimization by protease treatment to reduce the risk of rejection [11].

### 4.4. Species Compatibility in Regenerative Function

It is imperative that the ECM topology of the donor is matched to the recipient microenvironment. For instance, the helical fiber bundle structure of sheep rotator cuff tendon facilitates directional regeneration, while the parallel alignment structure of the rat caudal tendon is only suitable for simple stretching models [48,78]. It has been demonstrated that the combination of bovine decellularized scaffolds with human tendon stem cell ECM (tECM-DBTS) results in the up-regulation of Scleraxis and Tenomodulin expression, while porcine-derived scaffolds promote fibrocartilaginous differentiation to a greater extent, attributable to variations in TGF-β3 concentrations [11,48]. Notably, the equine tendon-specific COMP protein (cartilage oligomeric matrix protein) enhanced tendonogenic differentiation of BMSCs, but was retained by less than 50% during decellularization in rodents [11,79].

In summary, donor selection requires comprehensive consideration of the stability of ECM components, biomechanical fitness, microstructural similarity and immunocompatibility of target species. Bovine and porcine tendons are frequently favored due to their analogous ECM composition and mechanical properties to humans; nevertheless, the feasibility of cross-species application must be enhanced by pathological model screening and compositional optimization (e.g., GAG removal or collagen cross-linking enhancement) [11,80,81].

## 5. Applications of Decellularized Tendon Biomaterials

The decellularized tendon extracellular matrix (tECM) has been shown to function as a bioactive molecular carrier, exhibiting unique compositional features that include collagen, glycosaminoglycans, and endogenous growth factors (IGF-1, TGF-β, etc.). These factors have been demonstrated to promote tendon-specific regeneration through the modulation of Wnt/β-catenin and MAPK signaling pathways [11,82,83]. Advances in technology have facilitated the processing of tECM (in powder, gel, sheet and 3D-printed scaffold formats), with significant progress being made in the domains of anti-adhesion, mechanical enhancement, and immunomodulation [84,85,86]. Nevertheless, its clinical application remains constrained by fundamental issues, including the presence of immunogenic residues, the absence of standardization for large-scale production, and the need for enhanced personalization [8,87].

### 5.1. tECM Scaffolds

tECM scaffolds possess a microenvironment, structure, and functionality similar to those of native tendons, and they gradually degrade as tissue regeneration progresses [88]. tECM scaffolds in different forms can be applied to various scenarios to meet therapeutic requirements and personalized needs. Tao et al. [17] addressed the common issue of adhesion following tendon injuries by optimizing the decellularization method for bovine Achilles tendons using microsectioning techniques. This approach allowed the completion of the decellularization process within a shorter timeframe and with fewer reagents, successfully producing a decellularized tendon matrix (DTM) with excellent biosafety. After twelve weeks of subcutaneous implantation in mice, the DTM was completely degraded without causing significant inflammatory responses. In a rabbit xenotransplantation experiment, the DTM effectively prevented Achilles tendon adhesion and improved the quality of tendon repair. Huang et al. [89] developed a load-bearing tendon substitute, the BioTenoForce scaffold, based on tECM-derived gelatin methacrylate (GelMA). The core–shell structure of the scaffold enhanced interface binding strength, with its peel strength and peak load significantly surpassing those of other non-adhesive scaffolds. It demonstrated excellent biocompatibility and effectively promoted the differentiation of human adipose-derived stem cells into tendon-lineage cells in vivo. In an animal model of tendon injury, the implantation of BioTenoForce resulted in regenerated tissue exhibiting an organized, wavy matrix structure similar to that of native tendons. Zhao et al. [84] developed a decellularized tendon scaffold (DTS-TA) modified with tannic acid (TA) to address the issue of inflammation during tendon repair. The scaffold exhibited excellent biocompatibility, antioxidative, and anti-inflammatory properties. It effectively alleviated inflammation caused by tendon injury, eliminated excessive reactive oxygen species, and promoted tendon regeneration, providing a promising new scaffold material for tendon injury repair. In order to develop functional composite scaffolds, researchers successfully constructed a multilayered tECM-DBTS scaffold with synergistic biomechanical and biochemical functions by combining decellularized bovine tendon sheet (DBTS) with tendon stem cell-derived ECM (tECM). The unique structure of tECM-DBTS significantly enhances the efficiency of directional migration of stem cells and the regenerative ability of tendon [11,82]. In order to simulate the stem cell microenvironment accurately, decellularized tendon sections (DTS) have been shown to enhance the efficiency of tendonogenic differentiation of MSCs by preserving the ECM topology with growth factors (e.g., TGF-β3) [82,90]. Optimization strategies for xenograft immunogenicity have the potential to enhance the long-term clinical outcome of xenotendon grafts. This may be achieved through a post-decellularization modification strategy that preserves the endogenous capacity of the cells for collagen synthesis and masks antigenic epitopes in the extracellular matrix [85]. It is notable that decellularized tendon ECM from freshwater fish (e.g., tilapia) has demonstrated unique advantages, including low biological risk properties, the absence of religious or ethical constraints, and sustainable accessibility. This provides a potential alternative to xenografts [91].

The clinical translation of tECM scaffolds is still encumbered by numerous technical challenges. A primary concern pertains to the uncertainty surrounding immune responses. While decellularization has been demonstrated to reduce immunogenicity, the presence of residual proteoglycans and elastin can potentially trigger delayed-type hypersensitivity reactions, thereby compromising graft safety [85]. The bottleneck of standardized production is also in need of a breakthrough. The variability in the ECM composition of tendons from different species and anatomical sites has a significant impact on the current decellularization process, which lacks a uniform quality control standard. This results in fluctuations in performance from batch to batch [10]. The challenge of bioactivity retention further restricts scaffold functionality. Conventional physicochemical decellularization processes are susceptible to the destruction of pivotal signaling molecules, such as FGF-2, within the ECM. Consequently, there is an imperative for the development of less aggressive decellularization treatments that can preserve bioactivity [92]. Moreover, the absence of personalized design in existing stents restricts their clinical application, and the structure is challenging to adapt to the anatomical variations and dynamic mechanical needs of patients [93].

### 5.2. Tendon-Derived Hydrogels

Hydrogels are cross-linked network materials with high hydrophilicity. dECM is processed into pre-gel through freeze-drying, grinding, enzymatic digestion, and neutralization, forming a hydrogel at physiological temperature. Hydrogels exhibit excellent biocompatibility and biodegradability, providing a growth environment similar to natural ECM for cells. They can also serve as carriers for cells or drugs, accelerating tendon repair after injury [12]. Compared to other solid scaffolds, hydrogels have the advantages of injectability and adaptability to irregular defect shapes [94]. Lee et al. [95] developed an adhesive decellularized hydrogel modified with catechol groups under alkaline conditions to promote oxidative cross-linking. This approach significantly improved tissue adhesion and enhanced mechanical properties. Ning et al. [16] prepared a decellularized tendon hydrogel (T-gel) using macaque Achilles tendons and evaluated its microstructure, water absorption equilibrium, retained bioactive factors, and in vitro cytotoxicity. T-gel exhibited biocompatibility similar to that of commercial collagen gel (C-gel). Compared to C-gel, T-gel maintained a nanofiber structure and retained more bioactive factors, such as stromal cell-derived factor-1 (SDF-1) and fibromodulin (Fmod), within the tendon ECM microenvironment. Furthermore, T-gel not only promoted the proliferation of macaque tendon-derived stem cells (mTDSCs) but also significantly enhanced their migration ability and differentiation into tendon cells. Furthermore, a therapeutic study on tendinopathy utilized an injectable tendon decellularized extracellular matrix (tdECM) hydrogel. This hydrogel was found to provide a more cytocompatible microenvironment in comparison to a collagen hydrogel, while retaining the bioactive factors of the natural tendon ECM. The tdECM hydrogel demonstrated significant synergistic modulation of inflammation, promotion of M2 macrophage polarization, and enhancement of tendon regeneration and repair efficacy [96]. In a study of the use of tissue-specific hydrogels in microfluidic devices for three-dimensional culture of stem cells, the physicochemical properties of tdECM hydrogels were characterized, and the compatibility of tdECM hydrogels with microfluidic devices was demonstrated [69]. Wound healing and repair often require extended periods, leading researchers to explore various materials and methods to shorten the healing time after injury. Hydrogels play an important role in wound healing, including regulating inflammation, preventing infection, promoting tissue regeneration, and removing wound exudates [97]. Alkhilani et al. [98] created skin wounds in a rabbit model to evaluate the repair effects of tendon-derived hydrogels. The results showed that using tendon-derived hydrogels for wound treatment significantly promoted skin wound healing by providing growth factors and ECM needed for cells and tissues. It has been demonstrated by other studies that porcine Achilles tendon decellularized ECM is modified using microbial transglutaminase (MTG) to produce a cross-linked variant of ECM (MTG-ECM). The results demonstrated that MTG-ECM impeded the transition from type III to type I collagen in the wound area, which may result in a reduction in the development of wound scars [99].

Despite recent advances, technical bottlenecks and limitations still exist in the innovation and application of hydrogels. The primary constraint pertains to the inadequate mechanical strength exhibited by natural ECM hydrogels, which are unable to satisfy the mechanical demands associated with highly dynamically loaded tissues, such as tendons [100]. The ambiguity of the immunoregulatory mechanisms is also evident. The long-term regulation of the local immune microenvironment by hydrogel degradation products remains to be elucidated, particularly with regard to the absence of molecular mechanisms at the level of advanced technologies, such as single-cell sequencing [86,101]. The issue is further compounded by the absence of personalized adaptation, necessitating enhancement of the congruence between the rheological properties of the hydrogel and the anatomical characteristics of the injection site. The development of bespoke viscoelastic parameters tailored to individual patients is a prospective avenue for future research [102].

### 5.3. Bioinks

Three-dimensional (3D) bioprinting combines 3D printing technology with bioinks, commonly using modes such as inkjet printing, extrusion printing, and light-assisted printing. Bioinks can create highly biomimetic, biologically functional 3D structures layer by layer, such as artificial organs and tissue-engineered scaffolds. In such 3D structures, bioinks are precisely positioned at different levels to mimic the transition zones between body tissues. By controlling the composition and distribution of bioinks, smooth transitions between tissues can be achieved, better simulating and promoting tissue regeneration and repair [18,103]. Histologically, the tendon-bone interface (TBI) consists of three continuous regions: bone tissue, calcified and uncalcified fibrocartilage, and tendon tissue. In different tissue regions, the ECM composition and cell phenotypes exhibit gradient variations [21,104]. Chae et al. [105] developed bioinks from tECM and used 3D bioprinting to fabricate TBI patches. The TBI patches possess a 3D spatial gradient structure that mimics the transitional region of the tendon-bone interface, providing an appropriate microenvironment for cells, promoting the differentiation of human bone marrow mesenchymal stem cells, and significantly accelerating TBI repair and healing. Furthermore, studies have been conducted that target the TBI gradient mineralization interface using multi-material synergistic printing for prosthetic treatment. In this method, dual printheads print hydroxyapatite-containing bone ECM inks and TGF-β-containing tendon ECM inks, respectively. This bioprinting method has been shown to improve the mechanical properties and tissue integration of the TBI, as well as enhance angiogenesis and ECM formation [21]. It is important to note that dECM itself has inherent limitations, such as weak mechanical properties and poor printability. In order to obtain mechanically stable and fully cell-aligned structures, a highly concentrated bioink devoid of cells is utilized to support the mechanical properties, and a stable tissue structure is constructed by tracer flow to fully align the cells in a thin layer of cell-containing bioink. This cell-containing hybrid structure has the potential to serve as an efficient platform for musculoskeletal tissue alignment [106]. The process of printing flexible tissues with dECM bioinks is susceptible to deformation, and the incorporation of additional materials to enhance stiffness can impact bioactivity. Utilizing gelatinized dECM (GeldECM) as a rheology modifier, hybrid bioinks containing GeldECM undergo multiple cross-linking processes (including pre-cross-linking, visible light photocross-linking, and thermal cross-linking) both before and after printing. This approach is intended to enhance the stiffness and stability of the bioink while preserving bioactivity. The printed tissues demonstrate resilience to external vibrations and maintain their structural integrity. This provides a new idea for the preparation of flexible tissues (e.g., tendons) with high toughness [107]. Rosa et al. [108] combined biomimetic fibers with tECM to prepare bioinks, constructing a 3D tendon in vitro microphysiological system. This system replicates the arrangement and phenotypes of core tendon cells and integrates with vascular tissue to enable efficient cell population interactions. The physiological and biochemical stimuli generated by 3D printing and tECM hydrogels effectively induce the differentiation of human adipose-derived stem cells into tendon cells, fully simulating the developmental stages of tendons in the body.

In conclusion, tECM bioinks have facilitated the development of new methodologies for the automated generation of tendon organ-on-a-chip models. This development has provided significant support for in-depth studies of tendon physiological and pathological mechanisms, as well as for the testing of new treatments for tendon diseases. Nevertheless, there are both breakthroughs and challenges to be addressed. The gel kinetic limitation is the primary challenge, and the contradiction between the shear-thinning property and extrusion stability of dECM inks is still not completely resolved [109]. The viability of cells is a crucial consideration that further restricts the application of functional constructs. The primary cause of cell mortality during the printing process has been identified as shear stress damage. This necessitates the development of innovative hardware solutions, such as low-shear micro-extrusion printheads [106]. At present, there is an absence of a standardization system at the system level, which acts as an impediment to clinical translation. The presence of substantial variations in core parameters, including dECM ink concentration, viscosity, and cell density, across diverse research laboratories, poses a significant challenge in the comparison of research outcomes on a horizontal basis and their subsequent replication on a large scale. A more critical issue is that of cost–benefit imbalance. The expense of personalized bioprinting systems is a significant challenge, far exceeding that of traditional transplantation surgery.

## 6. Conclusions and Future Outlook

The use of decellularized biomaterials has driven rapid advancements in the field of tissue injury repair. In the complex microenvironment of natural tissues, numerous challenges remain in achieving cell interactions and functional restoration in vivo. With multidisciplinary collaborations, decellularized scaffold biomaterials [110] (particularly biomimetic scaffold systems) show great potential for tissue and organ repair, including tissues such as bone [111], cartilage [112], muscle [113], skin [114], blood vessels [115], and tendons [116], as well as organs such as the heart [117], liver [118], lungs [119], and kidneys [120]. In recent years, there have been frequent reports of porcine kidney [121], heart [122], and liver [123] transplants. These studies further underscore the broad prospects for xenogeneic biomaterials in tissue injury repair. The field of biomaterials is confronted with a multitude of challenges, with fundamental issues centered on the equilibrium between cellular residues and ECM component depletion in decellularization processes [124]. Key scientific challenges include the degradation of mechanical properties following long-term implantation, particularly in weight-bearing tissue repair, which can result in scaffold structural collapse [125,126]; the precise detection and elimination of xenoimmunogenic substances (e.g., α-Gal antigens) [127]; and the heterogeneity of age-associated immune responses [128]. Future breakthroughs will be focused on multidisciplinary technological innovations, including the use of CRISPR gene editing to construct low-immunogenic bio-donors [129] and vascularized biomimetic scaffolds through 3D bioprinting [130]; the establishment of a standardized quality control system based on proteomics [131] and mechanistic [132] profiling to optimize the process parameters; and the expansion of the application of organ microarray technology in the pre-assessment of personalized scaffold performance [109,133].

In recent years, xenogeneic decellularized tendons derived from species such as mice, monkeys, pigs, cattle, and sheep have been widely reported and applied in various scenarios for tendon tissue injury repair. Due to limitations such as genetic evolutionary distance (mice), ethical concerns (monkeys), and pathogen risks (cattle and sheep), pigs offer unique advantages as experimental animals for human biomedical research and product development, including similarities to humans in genome annotation, physiology, anatomical structure, and metabolic processes. Additionally, their high fertility, large litter sizes, and short reproductive cycles make pigs an ideal option [134,135,136,137]. However, conventional experimental pigs still carry risks of pathogen transmission. Specific pathogen-free (SPF) pigs, or even germ-free (GF) pigs, established through sterile cesarean section, can eliminate potential infection sources and pathogens, providing experimental donors with well-defined genetic structures and microbial backgrounds for exploring xenogeneic decellularized tendons.

Previously, our team has successfully established an independently controlled SPF pig population and built the largest domestic platform for the cultivation and application of GF pigs [138,139]. These experimental pig resources have been used to develop preparation systems for porcine-derived biomaterials, such as pig skin and small intestinal submucosa, providing core raw materials for the standardized development and utilization of porcine-derived bioproducts. Currently, our team has optimized a rapid standardized decellularization method for tendons and systematically analyzed the differences in tendon phenotypes, biomechanics, and matrix composition across different developmental stages, microbial levels, and anatomical locations. This work provides methodological references for the preparation of porcine-derived decellularized tendons, fundamental data for the development of decellularized tendon biomaterials, and significant insights for tendon tissue injury repair and treatment.

## References

[1] Steinmann S., Pfeifer C.G., Brochhausen C., Docheva D. (2020). Spectrum of Tendon Pathologies: Triggers, Trails and End-State. Int. J. Mol. Sci..

[2] Xu Z., Xu W., Zhang T., Luo L. (2024). Mechanisms of tendon-bone interface healing: Biomechanics, cell mechanics, and tissue engineering approaches. J. Orthop. Surg. Res..

[3] Yang S.M., Chen W.S. (2020). Conservative Treatment of Tendon Injuries. Am. J. Phys. Med. Rehabil..

[4] Jo S., Calfee R.P. (2023). General Principles of Flexor Tendon Repair. Hand Clin..

[5] Hoskins T., Patel J., Choi J.H., Fitzpatrick B., Begley B., Mazzei C.J., Harrington C.J., Miller J.M., Wittig J.C., Epstein D. (2023). Mini-Open Achilles Tendon Repair: Improving Outcomes While Decreasing Complications. Foot Ankle Spec..

[6] Tang J.B., Lalonde D., Harhaus L., Sadek A.F., Moriya K., Pan Z.J. (2022). Flexor tendon repair: Recent changes and current methods. J. Hand Surg. Eur. Vol..

[7] Roth S.P., Erbe I., Burk J. (2018). Decellularization of Large Tendon Specimens: Combination of Manually Performed Freeze-Thaw Cycles and Detergent Treatment. Decellularized Scaffolds and Organogenesis, Methods in Molecular Biology.

[8] Jin Y., Sun Q., Ma R., Li R., Qiao R., Li J., Wang L., Hu Y. (2024). The trend of allogeneic tendon decellularization: Literature review. Cell Tissue Bank..

[9] Perez M.L., Castells-Sala C., Lopez-Chicon P., Nieto-Nicolau N., Aiti A., Farinas O., Casaroli-Marano R.P., Porta O., Vilarrodona A. (2021). Fast protocol for the processing of split-thickness skin into decellularized human dermal matrix. Tissue Cell.

[10] Anjum S., Li T., Saeed M., Ao Q. (2024). Exploring polysaccharide and protein-enriched decellularized matrix scaffolds for tendon and ligament repair: A review. Int. J. Biol. Macromol..

[11] Cui J., Ning L.J., Wu F.P., Hu R.N., Li X., He S.K., Zhang Y.J., Luo J.J., Luo J.C., Qin T.W. (2022). Biomechanically and biochemically functional scaffold for recruitment of endogenous stem cells to promote tendon regeneration. npj Regen. Med..

[12] Chen R., Chen F., Chen K., Xu J. (2024). Advances in the application of hydrogel-based scaffolds for tendon repair. Genes Dis..

[13] Dang R., Chen L., Sefat F., Li X., Liu S., Yuan X., Ning X., Zhang Y.S., Ji P., Zhang X. (2022). A Natural Hydrogel with Prohealing Properties Enhances Tendon Regeneration. Small.

[14] Jiang Y., Zhu C., Ma X., Fan D. (2024). Smart hydrogel-based trends in future tendon injury repair: A review. Int. J. Biol. Macromol..

[15] Kent R.N., Huang A.H., Baker B.M. (2024). Augmentation of Tendon and Ligament Repair with Fiber-Reinforced Hydrogel Composites. Adv. Healthc. Mater..

[16] Ning L.J., Zhang Y.J., Zhang Y.J., Zhu M., Ding W., Jiang Y.L., Zhang Y., Luo J.C., Qin T.W. (2021). Enhancement of Migration and Tenogenic Differentiation of Macaca Mulatta Tendon-Derived Stem Cells by Decellularized Tendon Hydrogel. Front. Cell Dev. Biol..

[17] Tao M., Liang F., He J., Ye W., Javed R., Wang W., Yu T., Fan J., Tian X., Wang X. (2021). Decellularized tendon matrix membranes prevent post-surgical tendon adhesion and promote functional repair. Acta Biomater..

[18] Wan H., Xiang J., Mao G., Pan S., Li B., Lu Y. (2024). Recent Advances in the Application of 3D-Printing Bioinks Based on Decellularized Extracellular Matrix in Tissue Engineering. ACS Omega.

[19] Alhaskawi A., Zhou H., Dong Y., Zou X., Ezzi S.H.A., Kota V.G., Abdulla M.H.A., Tu T., Alenikova O., Abdalbary S. (2024). Advancements in 3D-printed artificial tendon. J. Biomed. Mater. Res. Part B Appl. Biomater..

[20] Kiratitanaporn W., Guan J., Tang M., Xiang Y., Lu T.Y., Balayan A., Lao A., Berry D.B., Chen S. (2024). 3D Printing of a Biomimetic Myotendinous Junction Assisted by Artificial Intelligence. Biomater. Sci..

[21] Kim W., Kwon D.R., Lee H., Lee J., Moon Y.S., Lee S.C., Kim G.H. (2025). 3D bioprinted multi-layered cell constructs with gradient core-shell interface for tendon-to-bone tissue regeneration. Bioact. Mater..

[22] Rosset J., Olaniyanu E., Stein K., Almeida N.D., França R. (2024). Exploring the Frontier of 3D Bioprinting for Tendon Regeneration: A Review. Eng.

[23] Zhao J., Zhang D., Lan Q., Zhong G., Liu Y., Holwell N., Wang X., Meng J., Yao J., Amsden B.G. (2024). Tendon Decellularized Matrix Modified Fibrous Scaffolds with Porous and Crimped Microstructure for Tendon Regeneration. ACS Appl. Bio Mater..

[24] Naba A. (2024). Mechanisms of assembly and remodelling of the extracellular matrix. Nat. Rev. Mol. Cell Biol..

[25] Tu T., Shi Y., Zhou B., Wang X., Zhang W., Zhou G., Mo X., Wang W., Wu J., Liu W. (2023). Type I collagen and fibromodulin enhance the tenogenic phenotype of hASCs and their potential for tendon regeneration. npj Regen. Med..

[26] Asgari M., Latifi N., Heris H.K., Vali H., Mongeau L. (2017). In vitro fibrillogenesis of tropocollagen type III in collagen type I affects its relative fibrillar topology and mechanics. Sci. Rep..

[27] Sun M., Luo E.Y., Adams S.M., Adams T., Ye Y., Shetye S.S., Soslowsky L.J., Birk D.E. (2020). Collagen XI regulates the acquisition of collagen fibril structure, organization and functional properties in tendon. Matrix Biol. J. Int. Soc. Matrix Biol..

[28] Izu Y., Adams S.M., Connizzo B.K., Beason D.P., Soslowsky L.J., Koch M., Birk D.E. (2021). Collagen XII mediated cellular and extracellular mechanisms regulate establishment of tendon structure and function. Matrix Biol. J. Int. Soc. Matrix Biol..

[29] Antoniel M., Traina F., Merlini L., Andrenacci D., Tigani D., Santi S., Cenni V., Sabatelli P., Faldini C., Squarzoni S. (2020). Tendon Extracellular Matrix Remodeling and Defective Cell Polarization in the Presence of Collagen VI Mutations. Cells.

[30] Thorpe C.T., Birch H.L., Clegg P.D., Screen H.R. (2013). The role of the non-collagenous matrix in tendon function. Int. J. Exp. Pathol..

[31] Eisner L.E., Rosario R., Andarawis-Puri N., Arruda E.M. (2022). The Role of the Non-Collagenous Extracellular Matrix in Tendon and Ligament Mechanical Behavior: A Review. J. Biomech. Eng..

[32] Narayanan N., Calve S. (2021). Extracellular matrix at the muscle—Tendon interface: Functional roles, techniques to explore and implications for regenerative medicine. Connect. Tissue Res..

[33] Marqueti R.C., Durigan J.L.Q., Oliveira A.J.S., Mekaro M.S., Guzzoni V., Aro A.A., Pimentel E.R., Selistre-de-Araujo H.S. (2018). Effects of aging and resistance training in rat tendon remodeling. FASEB J. Off. Publ. Fed. Am. Soc. Exp. Biol..

[34] Beach Z.M., Bonilla K.A., Dekhne M.S., Sun M., Adams T.H., Adams S.M., Weiss S.N., Rodriguez A.B., Shetye S.S., Birk D.E. (2022). Biglycan has a major role in maintenance of mature tendon mechanics. J. Orthop. Res. Off. Publ. Orthop. Res. Soc..

[35] Xu X., Ha P., Yen E., Li C., Zheng Z. (2022). Small Leucine-Rich Proteoglycans in Tendon Wound Healing. Adv. Wound Care.

[36] Chen D., Smith L.R., Khandekar G., Patel P., Yu C.K., Zhang K., Chen C.S., Han L., Wells R.G. (2020). Distinct effects of different matrix proteoglycans on collagen fibrillogenesis and cell-mediated collagen reorganization. Sci. Rep..

[37] Smith R., Önnerfjord P., Holmgren K., di Grado S., Dudhia J. (2020). Development of a Cartilage Oligomeric Matrix Protein Neo-Epitope Assay for the Detection of Intra-Thecal Tendon Disease. Int. J. Mol. Sci..

[38] Tashjian R.Z., Zitnay J., Kazmers N.H., Veerabhadraiah S.R., Zelada A.C., Honeggar M., Smith M.C., Chalmers P.N., Henninger H.B., Jurynec M.J. (2024). Tenascin C deletion impairs tendon healing and functional recovery after rotator cuff repair. J. Orthop. Res. Off. Publ. Orthop. Res. Soc..

[39] Gaffney L.S., Davis Z.G., Mora-Navarro C., Fisher M.B., Freytes D.O. (2022). Extracellular Matrix Hydrogels Promote Expression of Muscle-Tendon Junction Proteins. Tissue Eng. Part A.

[40] Ishizaki Y., Wang J., Kim J., Matsumoto T., Maeda E. (2024). Contributions of collagen and elastin to elastic behaviours of tendon fascicle. Acta Biomater..

[41] Grant T.M., Thompson M.S., Urban J., Yu J. (2013). Elastic fibers are broadly distributed in tendon and highly localized around tenocytes. J. Anat..

[42] Kuniakova M., Novakova Z.V., Haspinger D., Niestrawska J.A., Klein M., Galfiova P., Kovac J., Palkovic M., Danisovic L., Hammer N. (2024). Effects of Two Decellularization Protocols on the Mechanical Behavior and Structural Properties of the Human Urethra. Int. J. Mol. Sci..

[43] Chen T.A., Sharma D., Jia W., Ha D., Man K., Zhang J., Yang Y., Zhou Y., Kamp T.J., Zhao F. (2023). Detergent-Based Decellularization for Anisotropic Cardiac-Specific Extracellular Matrix Scaffold Generation. Biomimetics.

[44] Pierantoni M., Sharma K., Kok J., Novak V., Eliasson P., Isaksson H. (2025). Quantification of 3D microstructures in Achilles tendons during in situ loading reveals anisotropic fiber response. Acta Biomater..

[45] Newton J.B., Weiss S.N., Nuss C.A., Darrieutort-Laffite C., Eekhoff J.D., Birk D.E., Soslowsky L.J. (2024). Decorin and/or biglycan knockdown in aged mouse patellar tendon impacts fibril morphology, scar area, and mechanical properties. J. Orthop. Res. Off. Publ. Orthop. Res. Soc..

[46] Hatami-Marbini H., Emu M.E. (2025). Role of sulfated GAGs in shear mechanical properties of human and porcine cornea. Exp. Eye Res..

[47] Fu Y., Zhou Y., Wang K., Li Z., Kong W. (2024). Extracellular Matrix Interactome in Modulating Vascular Homeostasis and Remodeling. Circ. Res..

[48] Ostadi Moghaddam A., Arshee M.R., Lin Z., Sivaguru M., Phillips H., McFarlin B.L., Toussaint K.C., Wagoner Johnson A.J. (2023). Orientation-dependent indentation reveals the crosslink-mediated deformation mechanisms of collagen fibrils. Acta Biomater..

[49] Shama K.A., Greenberg Z.F., Tammame C., He M., Taylor B.L. (2024). Diseased Tendon Models Demonstrate Influence of Extracellular Matrix Alterations on Extracellular Vesicle Profile. Bioengineering.

[50] Pei Y., Yang W., Tang K., Kaplan D.L. (2023). Collagen processing with mesoscale aggregates as templates and building blocks. Biotechnol. Adv..

[51] Sun M., Li H., Hou Y., Huang N., Xia X., Zhu H., Xu Q., Lin Y., Xu L. (2023). Multifunctional tendon-mimetic hydrogels. Sci. Adv..

[52] Chen W., Chen M., Chen S., Wang S., Huang Z., Zhang L., Wu J., Peng W., Li H., Wen F. (2025). Decellularization of fish tissues for tissue engineering and regenerative medicine applications. Regen. Biomater..

[53] van Hengel E.V.A., van der Laan L.J.W., de Jonge J., Verstegen M.M.A. (2025). Towards Safety and Regulation Criteria for Clinical Applications of Decellularized Organ-Derived Matrices. Bioengineering.

[54] Aron J., Bual R., Alimasag J., Arellano F., Baclayon L., Bantilan Z.C., Lumancas G., Nisperos M.J., Labares M., Valle K.D.D. (2024). Effects of Various Decellularization Methods for the Development of Decellularized Extracellular Matrix from Tilapia (*Oreochromis niloticus*) Viscera. Int. J. Biomater..

[55] Yang J., Xu Y., Luo S., Dang H., Cao M. (2022). Effect of cryoprotectants on rat kidney decellularization by freeze-thaw process. Cryobiology.

[56] Roth S.P., Glauche S.M., Plenge A., Erbe I., Heller S., Burk J. (2017). Automated freeze-thaw cycles for decellularization of tendon tissue—A pilot study. BMC Biotechnol..

[57] Charras G., Yap A.S. (2018). Tensile Forces and Mechanotransduction at Cell-Cell Junctions. Curr. Biol. CB.

[58] Hamilton A.G., Townsend J.M., Detamore M.S. (2022). Automated Decellularization of Musculoskeletal Tissues with High Extracellular Matrix Retention. Tissue Eng. Part C Methods.

[59] Mendibil U., Ruiz-Hernandez R., Retegi-Carrion S., Garcia-Urquia N., Olalde-Graells B., Abarrategi A. (2020). Tissue-Specific Decellularization Methods: Rationale and Strategies to Achieve Regenerative Compounds. Int. J. Mol. Sci..

[60] Willemse J., Verstegen M.M.A., Vermeulen A., Schurink I.J., Roest H.P., van der Laan L.J.W., de Jonge J. (2020). Fast, robust and effective decellularization of whole human livers using mild detergents and pressure controlled perfusion. Mater. Sci. Eng. C Mater. Biol. Appl..

[61] Arumugam P., Kaarthikeyan G., Eswaramoorthy R. (2024). Comparative Evaluation of Three Different Demineralisation Protocols on the Physicochemical Properties and Biocompatibility of Decellularised Extracellular Matrix for Bone Tissue Engineering Applications. Cureus.

[62] Zhang A.Y., Bates S.J., Morrow E., Pham H., Pham B., Chang J. (2009). Tissue-engineered intrasynovial tendons: Optimization of acellularization and seeding. J. Rehabil. Res. Dev..

[63] Xing S., Liu C., Xu B., Chen J., Yin D., Zhang C. (2014). Effects of various decellularization methods on histological and biomechanical properties of rabbit tendons. Exp. Ther. Med..

[64] Youngstrom D.W., Barrett J.G., Jose R.R., Kaplan D.L. (2013). Functional characterization of detergent-decellularized equine tendon extracellular matrix for tissue engineering applications. PLoS ONE.

[65] White L.J., Taylor A.J., Faulk D.M., Keane T.J., Saldin L.T., Reing J.E., Swinehart I.T., Turner N.J., Ratner B.D., Badylak S.F. (2017). The impact of detergents on the tissue decellularization process: A ToF-SIMS study. Acta Biomater..

[66] Ingram J.H., Korossis S., Howling G., Fisher J., Ingham E. (2007). The use of ultrasonication to aid recellularization of acellular natural tissue scaffolds for use in anterior cruciate ligament reconstruction. Tissue Eng..

[67] Ning L.J., Jiang Y.L., Zhang C.H., Zhang Y., Yang J.L., Cui J., Zhang Y.J., Yao X., Luo J.C., Qin T.W. (2017). Fabrication and characterization of a decellularized bovine tendon sheet for tendon reconstruction. J. Biomed. Mater. Res. Part A.

[68] Ning L.J., Zhang Y., Chen X.H., Luo J.C., Li X.Q., Yang Z.M., Qin T.W. (2012). Preparation and characterization of decellularized tendon slices for tendon tissue engineering. J. Biomed. Mater. Res. Part A.

[69] Bhatt A., Dhiman N., Giri P.S., Kasinathan G.N., Pati F., Rath S.N. (2022). Biocompatibility-on-a-chip: Characterization and evaluation of decellularized tendon extracellular matrix (tdECM) hydrogel for 3D stem cell culture in a microfluidic device. Int. J. Biol. Macromol..

[70] Chatterjee M., Muljadi P.M., Andarawis-Puri N. (2022). The role of the tendon ECM in mechanotransduction: Disruption and repair following overuse. Connect. Tissue Res..

[71] Lal M.R., Agrawal D.K. (2024). Chronic Adaptation of Achilles Tendon Tissues upon Injury to Rotator Cuff Tendon in Hyperlipidemic Swine. J. Orthop. Sports Med..

[72] Castells-Sala C., Perez M.L., Lopez-Chicon P., Lopez-Puerto L., Martinez J.I.R., Ruiz-Ponsell L., Aiti A., Madariaga S.E., Sastre S., Farinas O. (2023). Development of a full-thickness acellular dermal graft from human skin: Case report of first patient rotator cuff patch augmentation repair. Transpl. Immunol..

[73] Terek J.C., Hebb M.O., Flynn L.E. (2023). Development of Brain-Derived Bioscaffolds for Neural Progenitor Cell Culture. ACS Pharmacol. Transl. Sci..

[74] Nagelli C.V., Hooke A., Quirk N., De Padilla C.L., Hewett T.E., van Griensven M., Coenen M., Berglund L., Evans C.H., Muller S.A. (2022). Mechanical and strain behaviour of human Achilles tendon during in vitro testing to failure. Eur. Cells Mater..

[75] Lal L.P.M., Agrawal D.K. (2024). Hyperlipidemia Induced Pathological Changes with no Effect in Biomechanical Properties in the Achilles Tendon of Young Swine. J. Orthop. Sports Med..

[76] Shi J., Yao H., Chong H., Hu X., Yang J., Dai X., Liu D., Wu Z., Dang M., Fei W. (2024). Tissue-engineered collagen matrix loaded with rat adipose-derived stem cells/human amniotic mesenchymal stem cells for rotator cuff tendon-bone repair. Int. J. Biol. Macromol..

[77] Gonzalez-Quevedo D., Sanchez-Porras D., Garcia-Garcia O.D., Chato-Astrain J., Diaz-Ramos M., Campos A., Carriel V., Campos F. (2022). Nanostructured fibrin-based hydrogel membranes for use as an augmentation strategy in Achilles tendon surgical repair in rats. Eur. Cells Mater..

[78] Peniche Silva C.J., Muller S.A., Quirk N., De la Vega R.E., Coenen M.J., Evans C.H., Balmayor E.R., van Griensven M. (2022). Enthesis: Not the same in each localisation—A molecular, histological and biomechanical study. Eur. Cells Mater..

[79] Stanczak M., Kacprzak B., Gawda P. (2024). Tendon Cell Biology: Effect of Mechanical Loading. Cell. Physiol. Biochem. Int. J. Exp. Cell. Physiol. Biochem. Pharmacol..

[80] Ellingson A.J., Pancheri N.M., Schiele N.R. (2022). Regulators of collagen crosslinking in developing and adult tendons. Eur. Cells Mater..

[81] Salman Hamza A., Traef Ali Q., Hadi Farman R. (2023). The Healing Effect of Biodegradable Scaffolds Treated with Bone-Marrow Obtained Mesenchymal Stem Cells on Major Tendon Damage in the Dog as a Model. Arch. Razi Inst..

[82] Ning L.J., Cui J., He S.K., Hu R.N., Yao X., Zhang Y., Ding W., Zhang Y.J., Luo J.C., Qin T.W. (2022). Constructing a highly bioactive tendon-regenerative scaffold by surface modification of tissue-specific stem cell-derived extracellular matrix. Regen. Biomater..

[83] Itoh M., Imasu H., Takano K., Umezu M., Okazaki K., Iwasaki K. (2022). Time-series biological responses toward decellularized bovine tendon graft and autograft for 52 consecutive weeks after rat anterior cruciate ligament reconstruction. Sci. Rep..

[84] Zhao L.L., Luo J.J., Cui J., Li X., Hu R.N., Xie X.Y., Zhang Y.J., Ding W., Ning L.J., Luo J.C. (2024). Tannic Acid-Modified Decellularized Tendon Scaffold with Antioxidant and Anti-Inflammatory Activities for Tendon Regeneration. ACS Appl. Mater. Interfaces.

[85] Jin H., Kang Y., Gao H., Lin Z., Huang D., Zheng Z., Zhao J., Wang L., Jiang J. (2024). Decellularization-Based Modification Strategy for Bioactive Xenografts Promoting Tendon Repair. Adv. Healthc. Mater..

[86] Wang R.M., Mesfin J.M., Karkanitsa M., Ungerleider J.L., Zelus E., Zhang Y., Kawakami Y., Kawakami Y., Kawakami T., Christman K.L. (2023). Immunomodulatory contribution of mast cells to the regenerative biomaterial microenvironment. npj Regen. Med..

[87] Ma R., Gao X., Jin Y., Wang X., Li R., Qiao R., Wang X., Liu D., Xie Z., Wang L. (2024). Is there a duration-characteristic relationship for trypsin exposure on tendon? A study on anterior cruciate ligament reconstruction in a rabbit model. Front. Med..

[88] Liu Q.W., Huang Q.M., Wu H.Y., Zuo G.S., Gu H.C., Deng K.Y., Xin H.B. (2021). Characteristics and Therapeutic Potential of Human Amnion-Derived Stem Cells. Int. J. Mol. Sci..

[89] Huang S., Rao Y., Zhou M., Blocki A.M., Chen X., Wen C., Ker D.F.E., Tuan R.S., Wang D.M. (2024). Engineering an extracellular matrix-functionalized, load-bearing tendon substitute for effective repair of large-to-massive tendon defects. Bioact. Mater..

[90] Rao Y., Zhu C., Suen H.C., Huang S., Liao J., Ker D.F.E., Tuan R.S., Wang D. (2022). Tenogenic induction of human adipose-derived stem cells by soluble tendon extracellular matrix: Composition and transcriptomic analyses. Stem Cell Res. Ther..

[91] Liu Z., Yu M.Z., Peng H., Liu R.T., Lim T., Zhang C.Q., Zhu Z.Z., Wei X.J. (2022). Decellularized tilapia fish skin: A novel candidate for tendon tissue engineering. Mater. Today Bio.

[92] Ghosh S., Pati F. (2023). Decellularized extracellular matrix and silk fibroin-based hybrid biomaterials: A comprehensive review on fabrication techniques and tissue-specific applications. Int. J. Biol. Macromol..

[93] Gao X.D., Zhang X.B., Zhang R.H., Yu D.C., Chen X.Y., Hu Y.C., Chen L., Zhou H.Y. (2022). Aggressive strategies for regenerating intervertebral discs: Stimulus-responsive composite hydrogels from single to multiscale delivery systems. J. Mater. Chem. B.

[94] Yao Z., Qian Y., Jin Y., Wang S., Li J., Yuan W.E., Fan C. (2022). Biomimetic multilayer polycaprolactone/sodium alginate hydrogel scaffolds loaded with melatonin facilitate tendon regeneration. Carbohydr. Polym..

[95] An S., Jeon E.J., Han S.Y., Jeon J., Lee M.J., Kim S., Shin M., Cho S.W. (2022). pH-Universal Catechol-Amine Chemistry for Versatile Hyaluronic Acid Bioadhesives. Small.

[96] Li D., Li S., He S., He H., Yuan G., Ma B., Zhang Y., Yuan C., Liu Z., Deng Z. (2025). Restoring tendon microenvironment in tendinopathy: Macrophage modulation and tendon regeneration with injectable tendon hydrogel and tendon-derived stem cells exosomes. Bioact. Mater..

[97] Chen G., Wang F., Zhang X., Shang Y., Zhao Y. (2023). Living microecological hydrogels for wound healing. Sci. Adv..

[98] Alkhilani M.A., Hammoodi O.T., Emran H.A., Alhayani W.A. (2024). Impact of Using Processed Urinary Bladder Submucosa and Hydrogel Fabricated from Tendon on Skin Healing Process in Rabbits. Vet. Med. Int..

[99] You C., Zhang Z., Guo Y., Liu S., Hu K., Zhan Y., Aihemaiti S., Tao S., Chu Y., Fan L. (2024). Application of extracellular matrix cross-linked by microbial transglutaminase to promote wound healing. Int. J. Biol. Macromol..

[100] Hu J., Liu S., Fan C. (2023). Applications of functionally-adapted hydrogels in tendon repair. Front. Bioeng. Biotechnol..

[101] Salthouse D., Novakovic K., Hilkens C.M.U., Ferreira A.M. (2023). Interplay between biomaterials and the immune system: Challenges and opportunities in regenerative medicine. Acta Biomater..

[102] Kong F., Mehwish N., Lee B.H. (2023). Emerging albumin hydrogels as personalized biomaterials. Acta Biomater..

[103] Zhang X., Li K., Wang C., Rao Y., Tuan R.S., Wang D.M., Ker D.F.E. (2024). Facile and rapid fabrication of a novel 3D-printable, visible light-crosslinkable and bioactive polythiourethane for large-to-massive rotator cuff tendon repair. Bioact. Mater..

[104] Chen C., Shi Q., Li M., Chen Y., Zhang T., Xu Y., Liao Y., Ding S., Wang Z., Li X. (2022). Engineering an enthesis-like graft for rotator cuff repair: An approach to fabricate highly biomimetic scaffold capable of zone-specifically releasing stem cell differentiation inducers. Bioact. Mater..

[105] Chae S., Sun Y., Choi Y.J., Ha D.H., Jeon I., Cho D.W. (2021). 3D cell-printing of tendon-bone interface using tissue-derived extracellular matrix bioinks for chronic rotator cuff repair. Biofabrication.

[106] Kim D., Kim G. (2023). Bioprinted hASC-laden cell constructs with mechanically stable and cell alignment cue for tenogenic differentiation. Biofabrication.

[107] Han H., Kim M., Yong U., Jo Y., Choi Y.M., Kim H.J., Hwang D.G., Kang D., Jang J. (2024). Tissue-specific gelatin bioink as a rheology modifier for high printability and adjustable tissue properties. Biomater. Sci..

[108] Monteiro R.F., Bakht S.M., Gomez-Florit M., Stievani F.C., Alves A.L.G., Reis R.L., Gomes M.E., Domingues R.M.A. (2023). Writing 3D In Vitro Models of Human Tendon within a Biomimetic Fibrillar Support Platform. ACS Appl. Mater. Interfaces.

[109] Wang B., Barcelo X., Von Euw S., Kelly D.J. (2023). 3D printing of mechanically functional meniscal tissue equivalents using high concentration extracellular matrix inks. Mater. Today Bio.

[110] Ge F., Lu Y., Li Q., Zhang X. (2020). Decellularized Extracellular Matrices for Tissue Engineering and Regeneration. Adv. Exp. Med. Biol..

[111] Amirazad H., Dadashpour M., Zarghami N. (2022). Application of decellularized bone matrix as a bioscaffold in bone tissue engineering. J. Biol. Eng..

[112] Kim Y.S., Majid M., Melchiorri A.J., Mikos A.G. (2019). Applications of decellularized extracellular matrix in bone and cartilage tissue engineering. Bioeng. Transl. Med..

[113] Shapiro L., Elsangeedy E., Lee H., Atala A., Yoo J.J., Lee S.J., Ju Y.M. (2019). In vitro evaluation of functionalized decellularized muscle scaffold for in situ skeletal muscle regeneration. Biomed. Mater..

[114] Ventura R.D., Padalhin A.R., Park C.M., Lee B.T. (2019). Enhanced decellularization technique of porcine dermal ECM for tissue engineering applications. Mater. Sci. Eng. C Mater. Biol. Appl..

[115] Liu C., Gao H., Sun G., Jiang X., Song S., Zhang J., Shen J. (2023). Decellularized Scaffold-Based Artificial Vascular Patch for Porcine Vascular Repair. ACS Appl. Bio Mater..

[116] Ghazanfari S., Alberti K.A., Xu Q., Khademhosseini A. (2019). Evaluation of an elastic decellularized tendon-derived scaffold for the vascular tissue engineering application. J. Biomed. Mater. Res. Part A.

[117] Kerr C.M., Silver S.E., Choi Y.S., Floy M.E., Bradshaw A.D., Cho S.W., Palecek S.P., Mei Y. (2024). Decellularized heart extracellular matrix alleviates activation of hiPSC-derived cardiac fibroblasts. Bioact. Mater..

[118] Khajavi M., Hashemi M., Kalalinia F. (2021). Recent advances in optimization of liver decellularization procedures used for liver regeneration. Life Sci..

[119] Leiby K.L., Niklason L.E. (2022). Lung Tissue Engineering: Toward a More Deliberate Approach. ACS Biomater. Sci. Eng..

[120] Mallis P., Oikonomidis C., Dimou Z., Stavropoulos-Giokas C., Michalopoulos E., Katsimpoulas M. (2021). Optimizing Decellularization Strategies for the Efficient Production of Whole Rat Kidney Scaffolds. Tissue Eng. Regen. Med..

[121] Wang Y., Chen G., Pan D., Guo H., Jiang H., Wang J., Feng H., He S., Du J., Zhang M. (2024). Pig-to-human kidney xenotransplants using genetically modified minipigs. Cell Rep. Med..

[122] (2023). Porcine heart xenotransplanation in brain-dead decedents. Nat. Med..

[123] Lamm V., Ekser B., Vagefi P.A., Cooper D.K.C. (2022). Bridging to Allotransplantation-Is Pig Liver Xenotransplantation the Best Option?. Transplantation.

[124] Kollmetz T., Castillo-Alcala F., Veale R.W.F., Taghavi N., van Heeswijk V.M., Persenaire M., May B.C.H., Dempsey S.G. (2024). Comparative Analysis of Commercially Available Extracellular Matrix Soft Tissue Bioscaffolds. Tissue Eng. Part A.

[125] Qiao S., Peijie T., Nan J. (2024). Crosslinking strategies of decellularized extracellular matrix in tissue regeneration. J. Biomed. Mater. Res. Part A.

[126] Fang W., Yang M., Jin Y., Zhang K., Wang Y., Liu M., Wang Y., Yang R., Fu Q. (2023). Injectable Decellularized Extracellular Matrix-Based Bio-Ink with Excellent Biocompatibility for Scarless Urethra Repair. Gels.

[127] Zhu D., Jiang Z., Li N., Wang X., Ren L., Ye Y., Pan Y., Yang G. (2022). Insights into the use of genetically modified decellularized biomaterials for tissue engineering and regenerative medicine. Adv. Drug Deliv. Rev..

[128] Han J., Rindone A.N., Elisseeff J.H. (2024). Immunoengineering Biomaterials for Musculoskeletal Tissue Repair across Lifespan. Adv. Mater..

[129] Li X., Chen Z., Ye W., Yu J., Zhang X., Li Y., Niu Y., Ran S., Wang S., Luo Z. (2023). High-throughput CRISPR technology: A novel horizon for solid organ transplantation. Front. Immunol..

[130] Chen G.H., Sia K.C., Liu S.W., Kao Y.C., Yang P.C., Ho C.H., Huang S.C., Lee P.Y., Liang M.Z., Chen L. (2025). Implantation of MSC spheroid-derived 3D decellularized ECM enriched with the MSC secretome ameliorates traumatic brain injury and promotes brain repair. Biomaterials.

[131] Stone R.N., Pu X., Oxford J.T. (2024). Proteomic dataset for decellularization of porcine auricular cartilage. BMC Res. Notes.

[132] Kim Y.H., Cidonio G., Kanczler J.M., Oreffo R.O., Dawson J.I. (2025). Human bone tissue-derived ECM hydrogels: Controlling physicochemical, biochemical, and biological properties through processing parameters. Bioact. Mater..

[133] Li X., Shan J., Chen X., Cui H., Wen G., Yu Y. (2023). Decellularized diseased tissues: Current state-of-the-art and future directions. MedComm.

[134] Hurst D.J., Cooper D.K.C. (2024). Pressing ethical issues relating to clinical pig organ transplantation studies. Xenotransplantation.

[135] Kozlov M. (2022). Clinical trials for pig-to-human organ transplants inch closer. Nature.

[136] Garry D.J., Weiner J.I., Greising S.M., Sachs D.H., Garry M.G. (2023). Xenotransplantation and exotransplantation: Strategies to expand the number of donor organs. Xenotransplantation.

[137] Cooper D.K.C., Pierson R.N. (2023). Milestones on the path to clinical pig organ xenotransplantation. Am. J. Transplant. Off. J. Am. Soc. Transplant. Am. Soc. Transpl. Surg..

[138] Sun J., Zhong H., Du L., Li X., Ding Y., Cao H., Liu Z., Ge L. (2018). Gene expression profiles of germ-free and conventional piglets from the same litter. Sci. Rep..

[139] Zhang J., Shen Y., Yang G., Sun J., Tang C., Liang H., Ma J., Wu X., Cao H., Wu M. (2023). Commensal microbiota modulates phenotypic characteristics and gene expression in piglet Peyer’s patches. Front. Physiol..

